# *Sindora stipitata* (Detarioideae, Leguminosae), a new species from Thailand

**DOI:** 10.3897/phytokeys.100.25870

**Published:** 2018-06-21

**Authors:** Wilawan Promprom, Wannachai Chatan, Peerapol Saisaard

**Affiliations:** 1 Department of Biology, Faculty of Science, Mahasarakham University, Kantharawichai District, Maha Sarakham Province, 44150, Thailand; 2 Number 57, Village No. 3, Thung Kula Subdistrict, Tha Tum district, Surin Province, Thailand

**Keywords:** *Sindora*, Fabaceae, Nakhon Phanom Province, plant diversity, Thailand, taxonomy

## Abstract

*Sindora stipitata*, a new species in the subfamily Detarioideae (Leguminosae), collected from Nakhon Phanom Province, Thailand, is described and illustrated. The new species is morphologically similar to *S. leiocarpa* but differs in its smaller stature (3–5 m high), 6-foliolate paripinnate leaves, falcate persistent stipules, presence of a petal auricle, absence of a petal claw, stipitate ovary and capitate stigma. A key to the Thailand and Malesia species of *Sindora* is provided.

## Introduction

*Sindora* Miq. is a genus in the tribe Detarieae (Detarioideae: Leguminosae) (McKinder 2005). It consists of 18–20 species distributed in Southeast Asia, one in Africa (Larsen et al. 1984, Lock and Heald 1994, McKinder 2005), two species in the South Asia (Kumar and Sane 2003) and two species in China (Chen et al. 2010). Three species have been identified in Thailand. Of these, *S. siamensis* Teysmann ex Miq. is a very common species of dry deciduous dipterocarp forests and beach forests, especially the type variety (Larsen et al. 1984).

During floristic surveys in the years 2009 to 2016 in the northeast of Thailand, a specimen of *Sindora* was collected from the Phulangka National Park in Nakhon Phanom Province which, on further investigation, was found to be clearly different from the previously reported species. It closely resembled *S. leiocarpa* Backer ex de Wit from Malesia. This was confirmed after comparing it against the type and description of *S. leiocarpa* and is here described as a new species.

## Materials and methods

Morphological characters were studied based on living plants observed during a field trip in the northeast of Thailand in the years 2009 to 2016 and from dried herbarium specimens housed in BKF and K. The studies consulted all relevant taxonomic literature in Thailand and neighbouring countries. Measurements were made using a vernier caliper and were examined under a stereo dissecting microscope. The conservation status of the new species was evaluated based on the guidelines of the International Union for Conservation of Nature (IUCN 2017).

## Taxonomy

### Sindora stipitata

Taxon classificationPlantaeFabalesFabaceae

Chatan & Promprom sp. nov.

urn:lsid:ipni.org:names:60476588-2

Figs 1 , 2

#### Diagnosis.

*Sindora stipitata* is very similar to *S. leiocarpa* from Malesia, but it is easily distinguished by the following characters: a smaller stature (3-5 m high), 6-foliolate paripinnate leaves, falcate persistent stipules, presence of a petal auricle, absence of a petal claw, stipitate ovary and capitate stigma.

**Figure 1.**
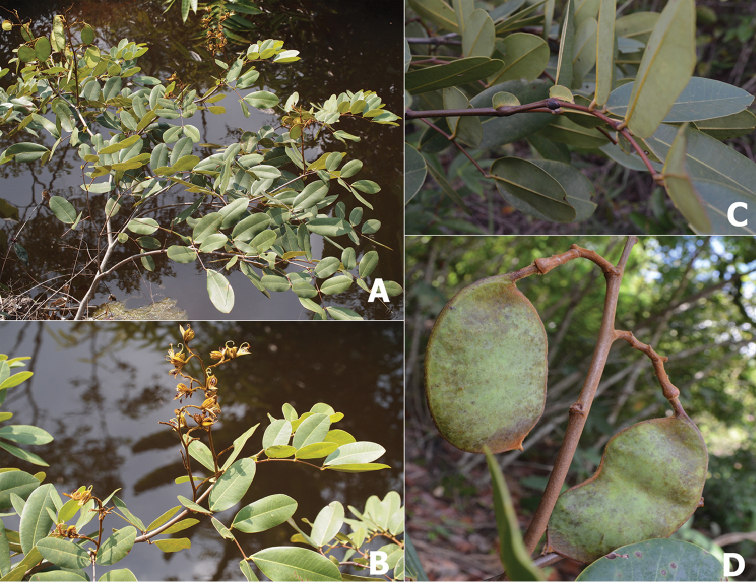
*Sindora stipitata*. **A** habit and habitat **B** branches and inflorescences **C** branch with leaves and stipules **D** branch with fruits. Photographs of the type specimen by W. Chatan.

**Figure 2.**
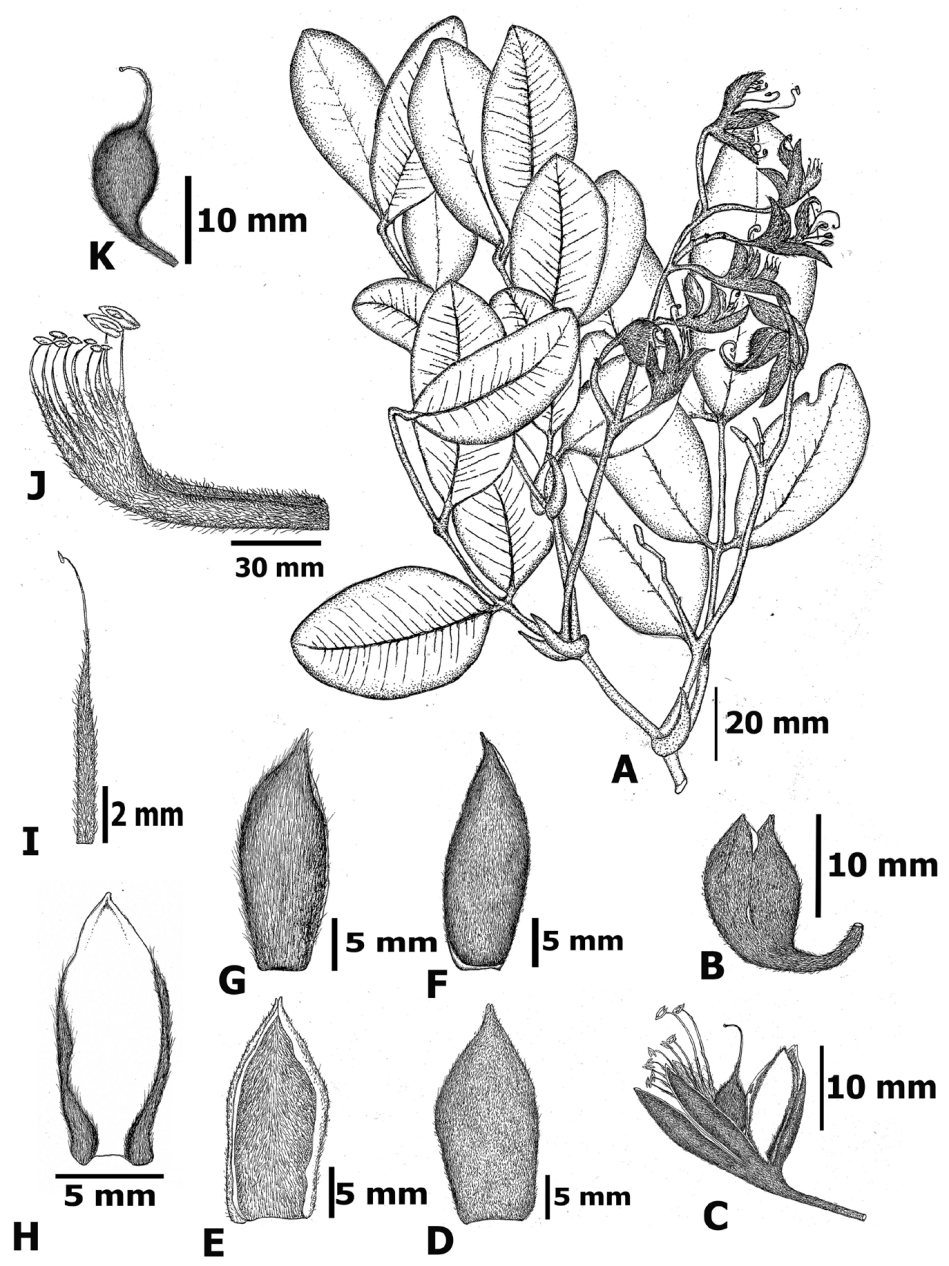
Line drawing of *Sindora stipitata* Chatan & Promprom, sp. nov. **A** a branch with leaves and inflorescence **B** floral bud **C** floret **D** posterior sepal (abaxial side) **E** posterior sepal (adaxial side) **F** one of the remaining narrower sepal (abaxial side) **G** one of the remaining narrower sepal (adaxial side) **H** petal (adaxial side) **I** free staminode **J** fused stamen **K** pistil. Illustration by W. Chatan (based on type specimen).

#### Type.

**Figure 3.**
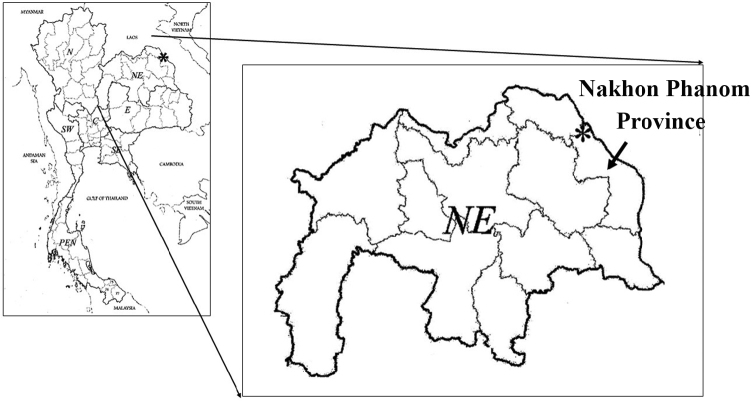
Distribution of *Sindora stipitata* Chatan & Promprom (*) in Phu Langka National Park, Nakhon Phanom Province, Thailand.

THAILAND. Nakhon Phanom Province: Phu Langka National Park, elevation 250–350 m, 17°59'18.7"N 104°07'50.1"E (Fig. 3), 20 April 2012, *W. Chatan 1231* (Holotype: BKF!; Isotype: K!).

#### Description.

Small tree, 3–5 m high. Stem diameter 3–5 cm. Stipules falcate, 23–25 × 1.0–1.3 mm, coriaceous, glabrous on both surfaces, persistent, venation distinctly reticulate. Leaves 6-foliolate, paripinnate, leaflets opposite; petioles 3.0–5.5 cm long, with sparse minute hairs or glabrescent; rachis 7–11 cm long, with sparse minute hairs or glabrescent. Leaflets rigidly coriaceous, elliptic or ovate or obovate or oblong, 7.5–11.5 × 3.5–5.3 cm; apex obtuse or sometime shallowly emarginate; base obtuse or cuneate, slightly asymmetric, glabrous on both surfaces, sometimes with a few minute hairs on the blade margin; abaxial side dull. Petiolules 3–6 mm long; glabrescent or with sparse minute hairs. Inflorescences paniculate, lax, up to 20 cm long, lateral branches up to 6 cm long, both rachises and lateral braches densely pubescent; bracts and bracteoles ca. 4.5 × 1.5 mm, puberulous; pedicels 11–12 mm long, densely puberulous. Buds ca. 15 × 10 mm, elliptic. Pedicel 10–11 mm long, densely puberulous. Hypanthium asymmetrically short and funnel-like, 0.5–1.0 mm long, brownish-yellow. Calyx lobes 4, thick, brownish-yellow, without any spiny outgrowth on the outer surface, densely puberulous outside, appressed hairs inside; the posterior lobe boat-shaped and obovate, 14–20 × 10–11 mm, apices acuminate; the other three lobes narrower, 15–20 × 4–6 mm, narrowly oblong or elliptic, apices narrowly acute. Corolla comprised of a solitary boat–shaped petal, lanceolate or narrowly obovate, thick, 15–17 × 5–6 mm, apices acute or acuminate, bases auriculate, puberulous outside, glabrous inside. Stamens 10, dorsifixed; upper stamen free, staminodal, 10–11 mm long, lower 2/3 of filament length densely puberulous, upper 1/3 of filament length sparsely hairy and glabrous near apex; remaining nine stamens, fertile, joined at the base into a sheath; sheath ca. 8–9 × 4.0–4.5 mm, densely puberulous on both surfaces; the two largest stamens are fertile, free parts of filament ca. 22–25 mm, lower half of the free parts of filament sparsely hairy and the upper half glabrous; anthers 5.5–6.0 × 1.5–1.6 mm, glabrous; other fertile stamens seven, free parts of filament 7–8 mm, lower 1/3 of the free parts densely puberulous, upper 2/3 of the free parts sparsely hairy and glabrous near apex; anthers, 2.5–3.0 × 1.0–1.2 mm, glabrous. Pistils with 5–6 mm stipe length; stipes densely puberulous; ovary asymmetrically elliptic; 7–8 × 4.5–5.0 mm, densely puberulous, no spiny outgrowth; style 9–11 mm long, densely puberulous on lower parts and sparsely hairy on the anterior side of upper parts; stigma capitate, glabrous. Pods circular or ellipsoid to obovate, 3.3–9.0 × 3.5–5.5 cm diameter, flattened, slightly smooth, unarmed, obscurely veined, with 1.5–5.0 mm long beaks, sparsely hairy on both surfaces, densely appressed hairs on the margin; seeds 1–4.

#### Other specimen examined.

THAILAND. Nakhon Phanom Province: Phu Langka National Park, 29 June 2013, fruiting, *P. Saisaard 55* (BKF).

#### Flowering and fruiting.

Flowering in March–May and fruiting April–June.

#### Distribution.

The new species is a Thai endemic and is known from only the type locality in the Phulangka National Park, Ban Pheang District, Nakhon Phanom Province, North-eastern Thailand.

#### Ecology.

This new species grows in open areas of dry deciduous forest at an elevation of 250–350 m.

#### Vernacular name.

Ma Kha Tae Nakhon Phanom, Mak Tae.

#### Etymology.

The specific epithet refers to its distinctly long ovary stipe. This character is one of many morphological characters that distinguishes the new species from its closely related species.

#### Preliminary conservation status.

*Sindora stipitata* is known only from the type locality and its estimated extent of occurrence is less than 100 km^2^. The number of mature individuals was less than 1,000 and the occupied area is continuing to decline slightly. Therefore, it should be considered as “Critically Endangered” according to the IUCN criteria B1 (IUCN 2017).

#### Discussion.

*Sindora stipitata* is closely related to *S. leiocarpa*, a plant that grows in Sumatra (Jimbi, Palembang and Riau) and Borneo (Sarawak, Brunei, Sabah and Kalimantan) (Hou 2000), but is easily distinguished from the latter by several morphological characters. Details of the differences between *S. stipitata* and *S. leiocarpa* are presented in Table 1.

The new species is also related to *S. coriacea* (Baker) Prain. Both have unarmed sepals, but *S. stipitata* is clearly different from *S. coriacea* by its stature of a small tree (3–5 m high) (vs. a large tree up to 50 m high), its abaxial leaflet surface dull brown (vs. shining), the two largest stamen 22–25 mm long (vs. ca. 10 mm long) and anthers 5.5–6 mm long (vs. 2–3 mm long). The new species also clearly differs from *S. laotica* Gagnep., a species that is distributed near the border of Thailand (in Vientiane, Phou Khao Khouay National Biodiversity Conservation Area, Lao) and Vietnam (Larsen, Larsen and Vidal 1980); the two species are different in that there is no spiny outgrowth on the outer surface of the calyx of the new species (vs. calyx spinescent). The following identification key for *Sindora* in Thailand and Malesia is constructed by modification of the key from the Flora Malesiana (Hou 2000).

**Table 1.** Distinguishing features between *S. stipitata* Chatan & Promprom and *S. leiocarpa* de Wit.

Characters	*S. stipitata* sp. nov.	*S. leiocarpa*
1. Habit	Small tree 3–5 m high, 3–5 cm diam.	Large tree, 25–45 m high, 45–80 cm diam.
2. Stipule	Falcate, 23–25 mm long	Lancelate, 3–5 mm long, caducous
3. Leaflet size	7.5–11.5 × 3.5-5.3 cm	2.5–9.5 × 2–5 cm
4. Pedicel length	10–11 mm	1–1.5 mm
5. Sepal size	14–20 × 4-11 mm	2–6 × 2–3.5 mm
6. Petal length	15–17 mm	5–6 mm
7. Petal claw	Absent	Present at about the lower half
8. Petal auricle	Present	Absent
9. Staminal sheath	8–9 mm long (high)	2–3 mm long (high)
10. Free filament part length of united stamens	7–25 mm	ca. 7 mm
11. Anther length of two largest stamens	22–25 mm	2–2.5 mm
12. Anther length of seven smallest fertile stamens	7–8 mm	Up to 0.75 mm
13. Ovary stipe length	5–6 mm	Subsessile or shortly stipitate (ca. 1 mm)
14. Ovary length	7–8 mm	ca. 4.5 mm
15. Stigma	Capitate	Obscure

### Key to *Sindora* Species in Thailand and Malesia

**Table:** 

1	Leaflets minutely puberulous or pubescent on both surfaces	***S. siamensis***
–	Leaflets glabrous on both surfaces or minutely puberulous or pubescent on the lower surface or rarely sparsely puberulous above	**2**
2	Leaflets glabrous on both surfaces	**3**
–	Leaflets only minutely puberulous or pubescent on the lower surface or rarely sparsely puberulous above	**8**
3	Apex of leaflet obtuse, rounded, retuse or emarginate	**4**
–	Apex of leaflets acute to acuminate	**6**
4	Calyx lobes densely spinescent	***S. supa***
–	Calyx lobes smooth, unarmed	**5**
5	Petal auricle present, petal claw absent, ovary stipe 5-6 mm long	***S. stipitata***
–	Petal auricle absent, petal claw present, ovary subsessile or shortly stipitate	***S. leiocarpa***
6	Midrib on the lower surface of the leaflet without a gland near the tip	***S. inermis***
–	Midrib on the lower surface of the leaflet with a gland near the tip	**7**
7	Midrib narrow and shallowly grooved on the upper surface	***S. coriacea***
–	Midrib slightly prominent on upper surface	***S. galedupa***
8	Fertile stamens 9, the uppermost staminode absent	***S. javanica***
–	Fertile stamens and staminodes 10, the uppermost staminode present	**9**
9	Calyx lobes with spinescent outgrowths or warts	**10**
–	Calyx lobes smooth, unarmed	**13**
10	Leaves 3-jugate	**11**
–	Leaves 4- or 5-jugate	**12**
11	Leaflets with apex acute or acuminate	***S. sumatrana***
–	Leaflets with apex obtuse, the tip slightly notched	***S. echinocalyx***
12	Calyx with spiny outgrowth mostly at the upper half	***S. wallichii***
–	Calyx with loosely, irregularly, minute, spiny outgrowth outside	***S. affinis***
13	Leaflets 3- or 4-jugate. Petals hairy on the longitudinal, central part	***S. beccariana***
–	Leaflets often 5- or 6-jugate. Petal glabous inside	**14**
14	Ovary glabous in the longitudinal, central part	***S. bruggemanii***
–	Ovary all densely woolly	**15**
15	Abaxial side of leaflet usually densely hirsute	***S. velutina***
–	Abaxial side of leaflet sparsely and minutely puberulous	***S. irpicina***

## Supplementary Material

XML Treatment for Sindora stipitata
